# Low-Cost Representative Sampling for a Natural Gas Distribution System in Transition

**DOI:** 10.1021/acsomega.2c05314

**Published:** 2022-11-23

**Authors:** Evan D. Sherwin, Ernest Lever, Adam R. Brandt

**Affiliations:** † Stanford University, Energy Science & Engineering, 367 Panama St. Room 49, Stanford, California 94305-4007, United States; ‡ R&D Director. GTI Energy, Energy Delivery. 1700 South Mount Prospect Road, Des Plaines, Illinois 60018-1804, United States. 847-544-3415; § Stanford University, Energy Science & Engineering. 066 Green Earth Sciences Bld. 367 Panama St., Stanford, California 94305-4007, United States. 650-724-8251

## Abstract

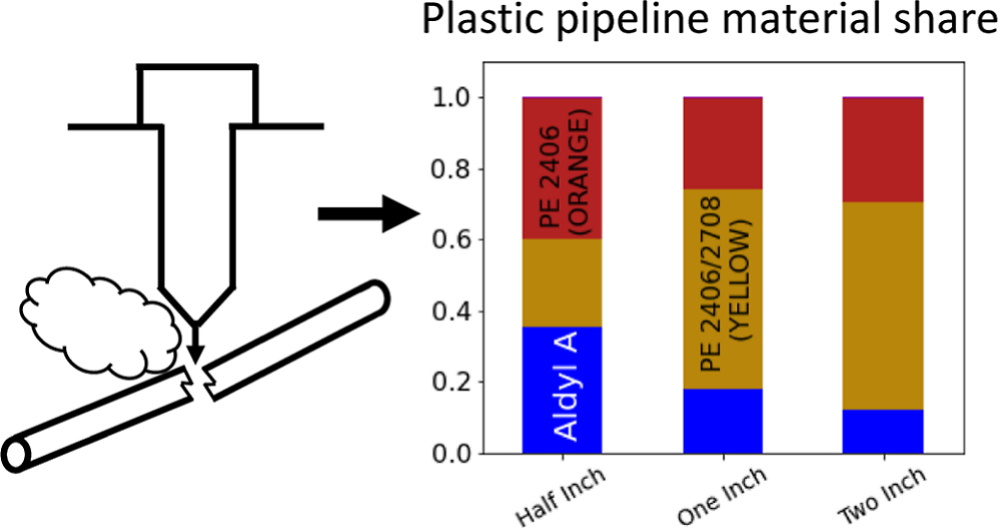

Natural gas distribution systems within municipalities supply a substantial fraction of energy consumed in the United States. As decarbonization of the natural gas system necessitates new modes of operation outside original design purposes, for example, increased hydrogen or biogas blending, it becomes increasingly important to understand in advance how existing infrastructure will respond to these changes. Such an analysis will require detailed information about the existing asset base, such as local soil composition, plastic type, and other characteristics that are not systematically tracked at present or have substantial missing data. Opportunistic sampling, for example, collecting measurements at assets that are already undergoing maintenance, has the potential to substantially reduce the cost of gathering such data but only if the results are representative of the full asset base. To assess prospects for such an approach, we employ a dataset including the entire service line and leak database from a large natural gas distribution utility (∼66,700 km of service pipelines and over 530,000 leaks over decades of observations). This dataset shows that service lines affected by excavation damage produce an approximately random sample of plastic and steel service lines, with similar distributions of component age, operating pressure, and pipeline diameter, as well as a relatively uniform spatial distribution. This means that opportunistic measurements at these locations will produce a first-order estimate of the relative prevalence of key characteristics across the utility’s full asset base of service lines. We employ this approach to estimate the plastic type, which is unknown for roughly 80% of plastic service lines in the database. We also find that while 32% of leaks across all components occur in threaded steel junctions, excavation damage accounts for 75% of hazardous grade 1 leaks in plastic service lines and corrosion accounts for 47% in steel service lines. Insights from this sampling approach can thus help natural gas utilities collect the data they need to ensure a safe and reliable transition to a lower-emission system.

## 1 Introduction

Natural gas distribution systems deliver 8.7% of nationally consumed energy to tens of millions of households and businesses in the United States through a network of over a million kilometers of natural gas service lines and mains.^1−3^ These networks were constructed over decades, with many containing assets that are more than 80 years old.^4^

A transition to a carbon-constrained world will likely require operating this network in new ways, potentially blending increasing amounts of hydrogen (H_2_) or biomethane or even retiring part or all of the system in favor of electrification of end uses.^5,6^ Any of these approaches will place stresses on the network outside of its original design purposes. How will existing steel and plastic assets interact with changing gas composition? How would operating a lower-throughput natural gas network affect system integrity, either with or without selective retirement of segments of the system? In any of these possible futures, how can we pre-emptively identify leak-prone assets to prevent emissions of climate-warming methane?

Answering these questions will often require detailed data about the material characteristics of assets such as pipelines, as well as the stresses these materials are under in situ. The in situ stresses are responsible for the evolution of damage in asset materials that will eventually lead to a variety of failure modes that compromise system safety and generate emissions. This damage evolution is strongly dependent on a significant number of interactions between the materials and the environment that are material-specific.^7^ In particular, increased blending of hydrogen or biomethane may cause additional embrittlement or corrosion, particularly in steel service lines, and may affect different types of plastic differently.^8,9^ More detailed knowledge of the actual materials present in the assets greatly reduces the uncertainty in assessing the likelihood of material degradation due to the environment and operating conditions. Similarly, plastic type is unknown for a substantial fraction of plastic service lines in at least some utilities. Hydrogen also has lower molar energy content than natural gas, which would require operation at higher pressures to maintain the same level of energy throughput. Similarly, renewable natural gas often has higher carbon dioxide content as well as siloxanes and other impurities, meaning that higher blending levels would require either expensive additional gas cleanup or a decline in the energy density and purity of pipeline natural gas.^10^

Recent empirical evidence suggests that natural gas distribution leaks are more prevalent and higher-emitting than previous estimates, highlighting the importance of system integrity management.^4^ Several studies have attempted to assess various aspects of natural gas pipeline system integrity using a variety of methods. Ahmed et al. assessed the effects of natural gas pipeline coatings on corrosion risk.^11^ Nykyforchyn et al. and Wasim et al. characterized risks of hydrogen embrittlement in steel pipelines.^9,12^ Chalgham et al. proposed a Bayesian prognosis and health monitoring-based approach to natural gas pipeline system integrity management aimed at reducing the likelihood of failure, including detailed mathematical treatment of the likelihood of asset failure due to corrosion and other mechanisms.^13^ However, existing studies of which we are aware have only limited access to the data needed to parameterize such models to simulate a given natural gas pipeline system.

Natural gas distribution utilities possess substantial databases of their existing assets and the history of those assets, including maintenance history and leak records. However, these databases were developed primarily for administrative reasons, rather than to inform engineering simulation models. In addition, some needed data are simply not available in bulk across the system due to historical loss of information or changes over time in recording practices. Thus, additional data collection will likely be required to enable the detailed analysis necessary to ensure the safe and environmentally sound operation of natural gas distribution systems in a period of transition.

Directly collecting in situ data from all underground distribution system assets would be prohibitively expensive and disruptive. Thankfully, in many cases, engineering feasibility studies require only information on the relative breakdown of key variables in the service territory, at least for a first pass to determine whether more detailed analysis is worthwhile. Broad system-level summary data can be acquired through statistical representative sampling of assets, with the requisite sample size depending on the application in question. Opportunistic measurements at underground assets during regularly occurring maintenance excavation would reduce the cost and disruption associated with collecting in situ measurements. However, assets that require maintenance may be in worse condition than the general population of assets, potentially introducing bias into this sampling approach.

This work proposes a method by which natural gas distribution utilities can take advantage of opportunistic measurements while averting this bias concern: collecting an approximately random sample of assets at locations of excavation damage. This approach allows utilities to collect necessary measurements of material quality, local soil composition, and more for a representative sample of assets, in this case, natural gas service pipelines, using the regularly occurring process of excavation damage. Such an approach can provide sufficient data to produce broad insights into questions about system integrity and leak prevention under a wide range of future operational strategies.

We present the first ever analysis in the peer-reviewed literature of which we are aware of the full service line and leak database of a natural gas distribution utility. Thus, both the opportunistic sampling method we introduce as well as the summary statistics of these databases provide new insights of interest to many stakeholders focusing on natural gas distribution system integrity. In addition, the basic approach we propose of sampling assets that must already be extracted due to damage can likely extend to other underground, underwater, or within-wall infrastructure systems for which extracting assets for measurement is difficult or costly.

## 2 Methods

### 2.1 Data

We analyze data from a large natural gas distribution utility describing their entire asset base of service pipelines, but not mains, as well as all historical leak records in their electronic database going back to the 1920s for pipelines and 1970s for leaks. The data are described in further detail in Supporting Information, Section S1.

### 2.2 Sampling Approach

We apply the following opportunistic sampling method to estimate the prevalence of key characteristics across the asset base: Collect desired measurements, for example, local soil composition and plastic type, at all locations of excavation damage across the utility or a subregion within the utility’s service territory. Compute the relative prevalence of these characteristics across the sampled assets, stratified by steel or plastic assets and across pipeline diameters. Extrapolate these stratified relative prevalence levels across the entire asset base (in this instance, service lines).

### 2.3 Assessing Representativeness

To determine the extent to which plastic service lines affected by excavation damage are representative of the full population of plastic service lines, we compare summary statistics across the two datasets. The most directly comparable fields across the databases of service lines and leaks are asset installation year, pressure rating, and pipeline diameter.

We also assess spatial representativeness across the two datasets in two ways. We perform a quantitative comparison of the number of excavation damage-related leaks per kilometer of service line installed across 13 divisions within the utility’s service territory covering 97% of total plastic service lines and 13 overlapping but not identical divisions covering 99.9% of steel service lines, described in Supporting Information, Section S2. We also qualitatively present the spatial distribution of plastic and steel excavation damage incidents across a densely populated 14 × 17.5 km region of the utility’s service territory.

Pipeline material composition and age varies widely across natural gas utilities. The results of this analysis are most representative of natural gas distribution utilities that have been in existence for 100 years or more and have roughly two-thirds plastic service lines and one-third steel service lines.

Note that the analysis presented in this study is only valid for the body of service lines and will not necessarily provide representative information about junctions or other types of assets, such as pressure regulators.

## 3 Results

Existing datasets provide the starting point for understanding the current state and future prospects of natural gas distribution system integrity. We leverage the full geo-referenced service line asset database for a large natural gas distribution utility, accounting for 66,993 km of mostly plastic and steel service lines, as well as the full leak database for the same utility, consisting of over 530,000 documented leaks from 1970 to early 2019. These databases have 119 and 159 features per record, respectively, summarized in Table 1 and in Supporting Information, Section S1.

**Table 1:** Selected Summary Statistics of all Service Lines and Documented Service Line Leaks in the Utility’s Territory^a^

	plastic	steel	all
total service lines	1,521,245	763,461	2,285,271
share of total	66.6%	33.4%	100%
average length [m]	29.31	29.33	29.32
total length [km]	44,586	22,390	66,993
median install year	1992	1956	1986
most common diameter	0.5 inches (85%)	0.75 inches (89%)	0.5 inches (57%)
plastic material reported	unknown (81%)	N/A	unknown (54%)
total leaks	62,878	57,367	129,609

a Categorical variables list the most common value and its frequency. Note that a small fraction of service line leaks occur in materials other than steel or plastic. See Supporting Information, Sections S4 and S5 for summaries of leaks from all assets as well as asset summary statistics.

Although plastic service lines represent 66.6% of the total, they constitute only 49% of service line leaks, while steels are 33.4% of total service lines and 44% of service line leaks. This indicates the importance of understanding system integrity for both types of materials. The remaining leaks occur in components other than service lines, including mains, transmission lines, risers, tee caps, valves, and taps. See Supporting Information, Section S4 for further details.

Notably, the precise plastic type, for example, Aldyl A, is not reported for 81% of plastic service lines. This is significant as there is documented variation in material integrity performance across plastics, with the commonly-used Aldyl A pipe prone to failure modes such as slow crack growth.^7^ The data do not track local soil composition. In addition, steel coating type data are missing for 46% of steel service lines, as shown in Supporting Information, Section S5.

Leaks can have a wide variety of causes and can vary in severity from grade 1, which indicates “an existing or probable hazard to persons or property and requires the operator to take action immediately to eliminate the hazard and make repairs,” to grade 3, indicating a leak that is “is nonhazardous at the time of detection and reasonably can be expected to remain nonhazardous”.^14^

**Figure 1:**
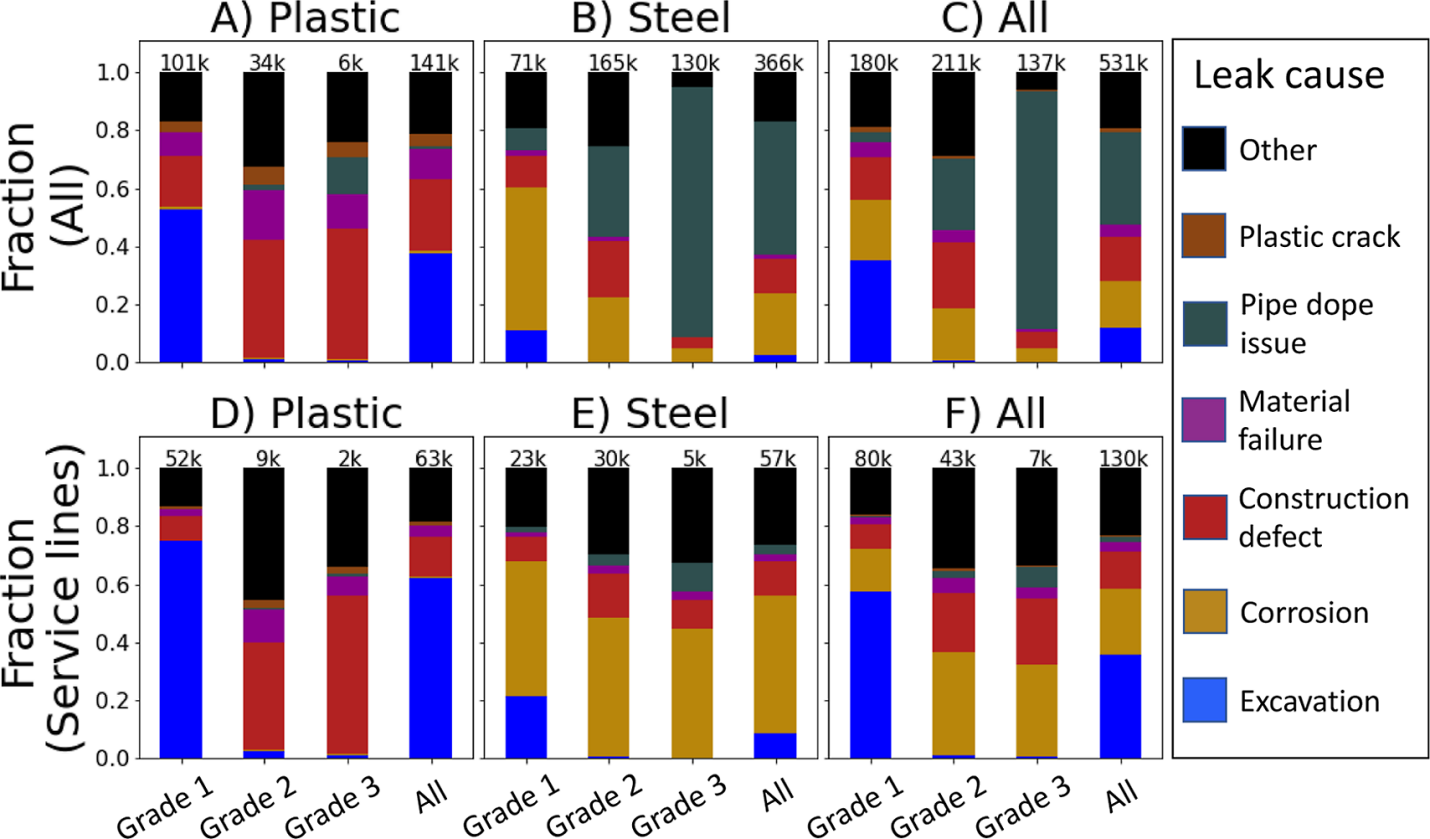
Breakdown of selected leak types by grade for the full leak database (A–C), also focusing on service lines (D–F). Total leak counts for each category are presented at the top of each bar. Grade 1 leaks pose the highest safety risk, while grade 3 pose the lowest. Excavation is the main cause of leaks in plastic service lines, while corrosion is the largest cause for steel service lines. Pipe dope issues are the most prevalent type of leak overall but typically are low-priority grade 3 leaks in riser threads. *“Excavation” uses the “Digin/Excavation” cause code from the database. Corrosion combines the “Atmospheric Corrosion” and “External Corrosion” cause codes. Grade 2 includes both grades 2 and 2+ (a now-discontinued subcategory of grade 2 leaks). See Supporting Information, Section S4 for all causes.

Figure 1 summarizes the fraction of leaks by grade for selected causes for all years in the database. Note that although excavation-related leaks are only 12% of all 530,812 leaks across all components, they represent 35% the 62,968 grade 1 leaks in the database and 75% of the 38,955 grade 1 leaks in plastic service lines. Leaks from external and atmospheric corrosion, which can be mitigated through proactive maintenance, account for 21% of all leaks in steel components and 47% of the 57,366 leaks in steel service lines. Leaks from construction defects, material failures, and plastic crack failures, which may also be preventable with targeted proactive maintenance, account for 19% of all leaks in service lines. Note that although pipe dope issues account for 32% of all leaks, these leaks tend to be less consequential grade 3 leaks and are concentrated in steel risers, not service lines. Results are qualitatively similar for distribution mains (see Supporting Information, Section S3). Thus, further insight into the current composition and state of repair of existing plastic and steel assets could help natural gas utilities mitigate risks associated with roughly 40% of their service line leaks. The remainder of the analysis focuses on leaks in service lines unless otherwise stated.

### 3.1 Representative Sampling via Excavation Damage

This work seeks to demonstrate that excavation damage affects an approximately random sample of service lines, providing the basis for a roughly statistically representative data collection approach to learn population-level characteristics. To do this, we compare the measured characteristics of assets affected by excavation damage with the general asset population. Excavation damage refers to damage to a utility asset caused by construction equipment, generally in the process of digging. Excavation damage can be inflicted by the utility during routine operations (first-party damage), by a contractor of the utility (second-party damage), or by an entity not working for the utility (third-party damage).

**Figure 2:**
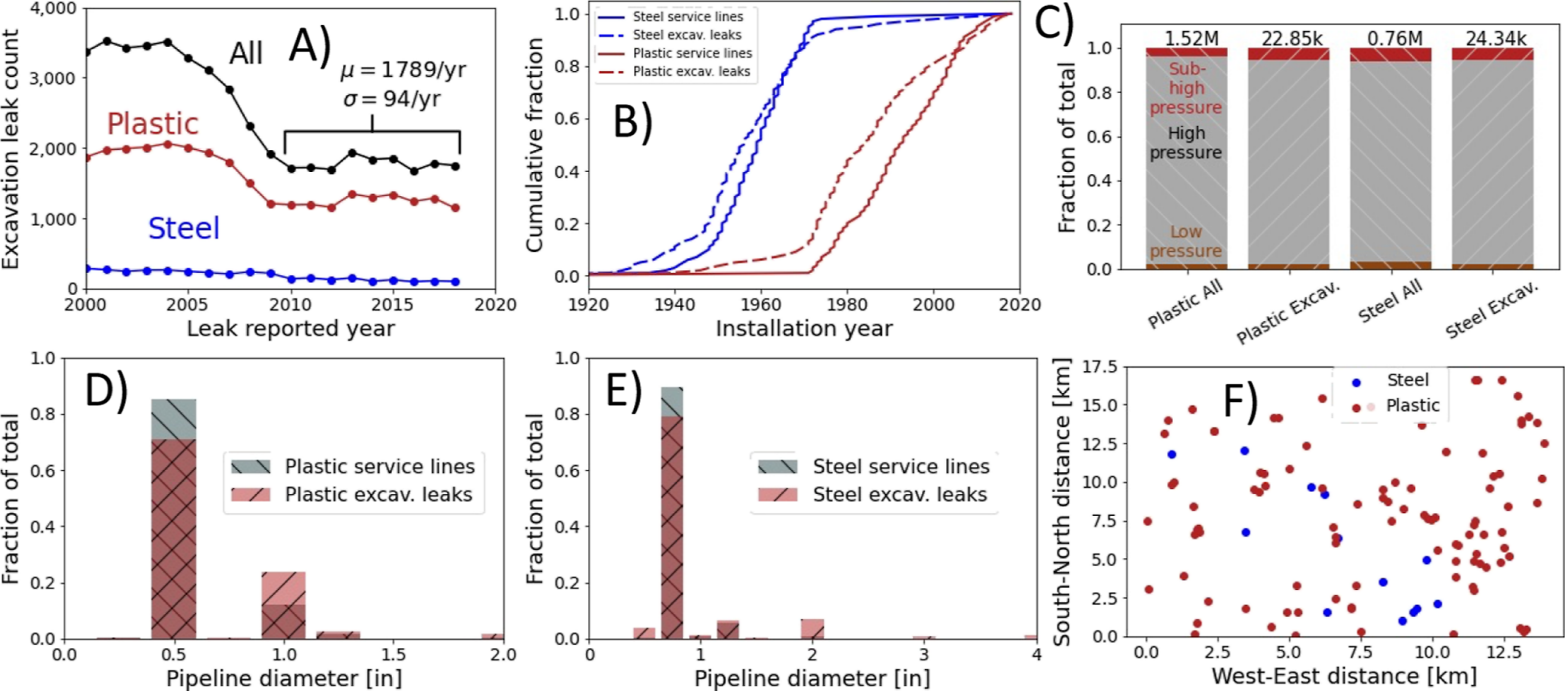
Comparison of service lines affected by excavation damage since 2009 with the full population of service lines. (A) Annual excavation damage leak incidence by pipeline material. Note that “All” includes leaks from assets other than service lines, while plastic and steel only include leaks from service lines. (B) Age distribution for all plastic and steel service lines as well as those affected by excavation damage (excludes assets with installation year before 1850). (C) Pressure rating and pipeline diameter for all (D) plastic and (E) steel service lines as well as lines affected by excavation damage. Hatching from top-left to bottom-right indicates all service lines, while hatching from bottom-left to top-right indicates only those affected by excavation damage. (F) Spatial distribution of excavation damage leak incidence in a densely populated example region of 14 × 17.5 km for plastic and steel service lines.

Leaks caused by excavation damage account for 12% of all recorded leaks and 5% of all leaks from 2009 through early 2019, when data end. Figure 2A demonstrates that rates of excavation damage have remained roughly constant between 1,674 and 1,940 per year since 2009, falling from a peak of 3,519 per year in 2001 following a damage prevention campaign. This suggests that excavation leak incidence rates have been relatively steady in the final 10 full years of analysis. For this reason, the remaining analysis of leak data will focus on the years 2009 on. Note that some excavation leaks do not affect service lines, hence the discrepancy between the sum of plastic and steel excavation leaks and the totals in black.

Figure 2B shows that the age distribution of plastic and steel service lines affected by excavation damage approximates the age distribution of the full population of assets in the database. The median installation year for all plastic service lines is 1992, falling to 1984 for plastic service lines affected by excavation damage, a statistically significant difference as discussed in Supporting Information, Section S6. This discrepancy between the two distributions remains within 10 years between the 25th and 100th percentiles of the age distributions. Because plastic service lines were installed over a period of roughly 50 years, as illustrated in Figure 2B, a difference of 8–10 years corresponds to approximately 16–20% error in the estimated median asset age. This gives a sense of the magnitude of error one can expect this method to introduce into key summary statistics. This level of error may be acceptable for some applications but may require more precise techniques for others.

Note that 9% of plastic and 24% of steel service line assets have invalid installation dates before the year 1850. Assets affected by excavation damage have a lower rate of missing installation dates, 2% for plastic and 2.5% for steel. Notably, the first percentile age of plastic service lines with valid installation years is 1971, while it is 1939 for those affected by excavation damage. This discrepancy in missing data proportions may explain some of the divergence between the two distributions. In particular, if the 9% of plastic service line asset records with invalid installation dates correspond to service lines installed before 1980, this would account for much of the observed gap between installation year distributions.

The statistically significant difference between median asset ages demonstrates that the proposed opportunistic sampling method does not produce a truly representative sample of the underlying assets, only an approximate sample. In this instance, our proposed approach is clearly a biased estimator of median plastic asset age, with a sample median that does not converge to the population median. However, biased estimators are widely used in statistics and machine learning. Total error in any estimator contains terms related both to bias and variance, as illustrated in the bias-variance tradeoff.^15^ Thus, our proposed method can still produce valuable insights, even if it introduces a quantifiable amount of error into key summary statistics. This is the basis upon which we say that our method produces an approximately representative sample of the population of assets.

The median installation year is 1959 for all steel service lines and 1956 for steel service lines affected by excavation damage, with the two distributions remaining within 5 years of each other from the 25th to the 92nd percentiles, with sharper divergences at the lower and higher percentiles. For older assets, this is largely due to a significant amount of missing data in the asset database (a missing installation year is often coded as the year 1800). This utility largely stopped installing new steel service lines in the late 1960s, as shown in Figure 2B. One possible explanation for the disproportionate number of newer steel service lines affected by excavation damage is that they may be in locations of higher excavation activity and thus more likely to be struck, replaced with a steel component, and struck again. We do not have data on the spatial and temporal extent of construction activity that would be necessary to assess this hypothesis in more detail.

Thus, the age distribution of both plastic and steel service lines is largely similar, though not identical, to the age distribution of all assets. The largest deviations occur for newer steel service lines (which represent only a few percent of the total) and older steel and plastic service lines, where the underlying age distribution is not well characterized for the full population. Because we do not know the true installation year for assets with a missing installation year, we cannot assess the extent to which this missing data issue introduces bias into our results. For plastic service lines, installation year is missing for only 8% of installed assets and 1% of those affected by excavation damage, limiting the potential magnitude of this effect. Installation year is missing for 23% of steel service lines and 2% of those affected by excavation damage, suggesting greater uncertainty in results for these assets.

Figure 2C demonstrates that the distribution of pressure ratings is comparable between steel and plastic service lines affected by excavation damage and their counterparts in the full population, with 90–95% rated for high pressure in all cases. Note that a small fraction of service line excavation damage records, 2% for plastic and 3% for steel, either have no pressure rating recorded or correspond to transmission assets. We exclude these from the above estimated pressure proportions as they are likely misclassified in the database. Figure 2D,E demonstrates that pipelines with larger diameter are moderately more likely to be affected by excavation damage, with 0.5-inch plastic service lines representing 85% of all plastic service lines but only 71% of those with excavation-related leaks. 1-inch plastic service lines make up the bulk of the remainder in both cases. Similarly, 0.75-inch service lines represent 89% of all steel service lines but only 79% of those with excavation-related leaks. This suggests that data collection at locations of excavation damage could stratify by pipeline diameter to more accurately learn underlying system characteristics.

Note that due to large sample sizes for both service line assets and excavation-related leaks, sample sizes are too large to meaningfully conduct traditional tests of statistical significance for the characteristics shown in Figure 2B–E. A *t*-test will show that even small differences in key characteristics between excavation-related leaks and service line assets are statistically significant. However, a benefit of this relatively large sample size is that differences observed between the full population of plastic and steel service lines and the subset of assets with excavation-related leaks give rough bounds on the expected deviation between the true population of assets and the “sample” collected through excavation-related leaks.

We assess spatial representativeness of excavation damage in two ways. Figure 2F shows the spatial distribution of excavation damage for plastic and steel service lines in a densely populated urban area of 14 × 17.5 km. The resulting spatial distribution is spread out across the full area, qualitatively suggesting that excavation damage is spread across the population of underlying assets. Unfortunately, this form of analysis can only give a qualitative indication of the spatial representativeness of excavation damage, in part because more than half of excavation-related leaks in the database from 2009 on are missing latitude and longitude coordinates, and asset location formats are not easily compatible between the leak and service line databases. Furthermore, using this dataset, we cannot account for fine-grained spatial variation in excavation damage due to increased local construction activity, which is often uneven within an urban area.

On a regional level, we assess spatial representativeness by comparing the number of excavation-related service line leaks per kilometer of plastic service line across the utility’s roughly 20 divisions listed in the database. Thirteen divisions with at least 1,000 km of plastic service lines account for 97% of all plastic service lines in the utility’s territory (by length). From 2009 through early 2019, these 13 divisions have between 0.019 and 0.47 excavation-related leaks in plastic service lines per kilometer of plastic service line installed. Of these, all but two are between 0.09 and 0.26 excavation-related leaks per kilometer of plastic service line. The remaining divisions, accounting for <3% of total plastic service lines, report substantially higher rates in some cases, but this appears to be due to internal accounting differences across databases rather than a true divergence in the rate of excavation damage.

Damage incidence rates are lower for steel service lines, between 0.002 and 0.309 service leaks per km from 2009 through early 2019 for 13 divisions with at least 500 km of steel service line (over 99.9% of the total). All but two of these divisions are between 0.01 and 0.06 leaks per km. See Supporting Information, Section S2 for further details. This suggests that despite some variability, rates of excavation damage for plastic service lines are generally within a factor of 2 and at most a factor of 5 across major divisions in the utility’s service territory, with somewhat higher variability for steel service lines. It may be necessary to correct for these modest variations in excavation damage incidence when generalizing results of excavation damage-based sampling to the full population of service lines in the utility’s service territory.

Thus, by the key metrics allowed by the data available, service lines affected by excavation damage appear, to first order, to be statistically representative of the full utility asset base in terms of installation year, pressure rating, and spatial distribution. Plastic and steel service lines should be treated separately and possibly segmented by pipeline diameter.

### 3.2 Opportunistic Sampling to Estimate Plastic Type

Leak records often include a more detailed description of the material type than asset records as the damaged asset is profiled extensively during the reporting process. As a result, if one accepts the above argument that excavation damage is an approximately representative sample of the existing asset base (accounting for the lower probability of puncture for steel service lines compared to plastics), we can estimate the distribution of plastic types, for example, Aldyl A or various forms of polyethylene, across the utility’s full asset base of 44,586 km of plastic service lines.

**Figure 3:**
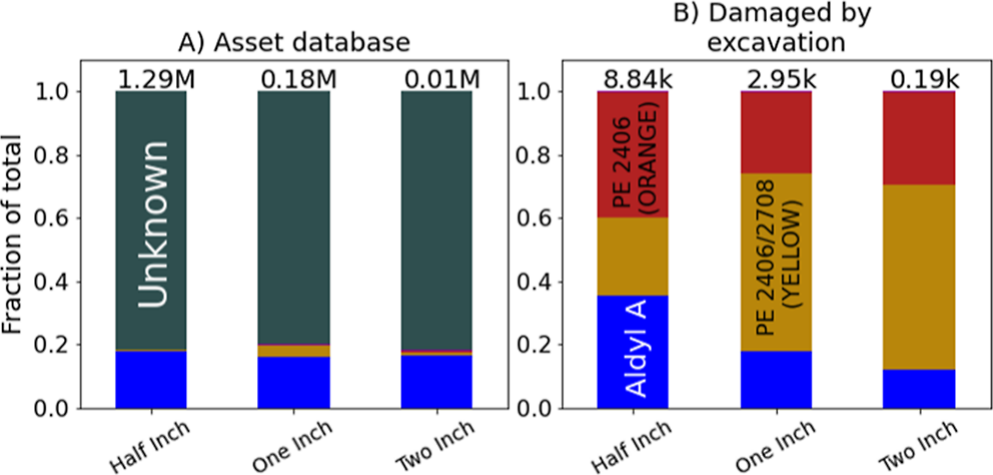
Plastic type for service lines. (A) In the utility’s asset database, plastic composition is unknown for roughly 80% of plastic service lines. (B) Breakdown by plastic type for assets affected by excavation damage since 2009, which our results suggest is an approximately random sample and thus approximately representative of the full population of plastic service lines. Total asset count listed above each bar.

Figure 3A shows the material composition breakdown of plastic service lines in the utility’s existing asset database, which is unknown for 80% or more of all half-inch, one-inch, and two-inch plastic service lines. 16–18% of these service lines are identified as Aldyl A, with other plastic types accounting for the remainder.

Figure 3B shows the same breakdown for plastic service lines with excavation-related leaks logged from 2009 onward. If excavation damage does indeed affect a representative sample of plastic service lines, after accounting for the pipeline diameter, then this should approximate the material composition for the full population of assets. Note that although Aldyl A is the most commonly identified material in the asset database, PE 2406 (orange) and PE 2406/2708 (yellow), both polyethylene materials, predominate here, accounting for 65–77% of plastic service lines affected by excavation damage depending on the pipeline diameter. This apparent large share of polyethylene service lines, which have somewhat different material properties than Aldyl A service lines, would remain unknown in the absence of an approximately random sampling approach such as that proposed here.

### 3.3 Analysis of Excavation Cost Savings

Given the speed of transition toward lower-carbon forms of energy, including in buildings, the costs of failing to adequately model the safety implications of large changes in the operational profile of natural gas distribution systems could be quite large but are difficult to quantify. As discussed earlier, such an analysis will likely require system-level data that utilities have not historically collected.

Suppose a utility wished to collect 1,000 measurements of underground assets in a year from truly random sites, many of which are below paved areas. The cost of repairing a residential underground water main is often roughly $5,000, while the cost of replacing a residence’s underground natural gas connection is estimated at $3,000–7,000.^16,17^ Neither of these estimates includes the cost of excavating asphalt and necessary safety equipment and personnel associated with road maintenance or the cost of temporarily shutting off gas service to nearby residences and businesses. Accounting for these costs, a single truly random measurement could easily reach $10,000 in costs borne both by the utility and by customers whose energy supply is disrupted. As a result, 1,000 measurements may cost as much as $10 million or more, not including the cost of the equipment and procedures necessary to conduct field or laboratory testing.

In contrast, opportunistic sampling at 1,000 of the roughly 1,800 annual locations of excavation damage-related leaks in the utility’s service territory would not incur any additional cost beyond the expenses necessary for measurement equipment and any laboratory testing. Thus, such an opportunistic sampling approach would provide the utility with the data needed to inform necessary engineering simulation modeling while saving as much as $10 million (or more) in excavation costs alone. Smaller utilities and utilities with lower rates of excavation damage may need to collect data over a longer period of time to achieve similar results. The precise sample size needed will depend on the application in question.

### 3.4 Key Uncertainties

The largest uncertainties introduced by this method relate to potential confounding factors in the underlying data-generating process of excavation damage to distribution pipelines. If excavation activity varies significantly in correlation with any aspect of system composition, this may introduce bias into estimates related to that aspect. For instance, if regions of high construction activity tend to have newer or older pipeline assets, this will result in estimates of asset age that are too low or too high on average, respectively. Improved construction activity data could help address this uncertainty.

Relatedly, if excavation damage is more frequent in one part of the system than another and the two regions have different asset compositions (e.g., more Aldyl-A plastic material in one region and more polyethylene in the other), this may result in overestimation of the prevalence of the materials that are more common in the area with greater incidence of damage. This uncertainty can be corrected by accounting across regions for frequency of excavation damage per unit pipeline length through some form of stratification.

Measurements of material quality based on assets affected by excavation damage must be conducted at sections of the asset to which damage has not propagated. Estimating material quality based on damaged sections of assets will likely oversample assets in worse condition. The distance of damage propagation depends on the asset material, the force of impact, and numerous environmental factors.

The possibility of nonrandom missing data patterns among either the asset or leak databases adds further uncertainty into our results. As noted earlier, if plastic service line asset records with missing data in fact correspond to older assets, this could explain much of the observed discrepancy in the age distributions for plastic service lines. If utilities are able to directly match leak records to asset records, which we cannot with the datasets available, it should be possible to answer key questions about service lines with missing installation year data.

Further analysis of similar utility asset and leak databases can further characterize key uncertainties related to this method, particularly if these datasets contain additional common fields between assets and leaks beyond those available in this paper. In addition, a utility implementing this method may opt to collect a small number of truly random asset samples to assist in calibration and uncertainty quantification of opportunistically collected samples.

## 4 Conclusions and Discussion

This study highlights a method that provides a statistically defensible pathway for natural gas distribution utilities to gain useful insight into new dimensions of their entire service pipeline asset base using opportunistic measurements. As the energy system transitions toward substantially lower greenhouse gas emissions, this will help integrity managers safely guide distribution systems into previously uncharted territory under operating conditions well outside initial design parameters.

One key factor for such opportunistic sampling is identifying a precipitating event that is approximately randomly distributed across the asset base. In this instance, data from plastic service lines affected by excavation damage are similar, at least to first order, to the full population of assets across all measured characteristics. This indicates that measurements collected at locations of excavation damage can provide first-order estimates of the population-level distribution of the measured characteristic in question.

This paper demonstrates this method in practice by estimating the precise plastic type used in plastic service lines in the utility’s service territory, which is not recorded for 81% of plastic service lines in the utility’s asset database. This information allows engineering simulation tools to more accurately model this important parameter, which otherwise would need to be based on zeroth-order assumptions, such as national averages or subjective judgments elicited from utility integrity management teams.

Relatively small additional data collection efforts on the part of natural gas distribution utilities, such as destructive lab testing of soil and pipeline samples collected at excavation damage locations (including an undamaged segment of the pipeline), could provide further critical information for integrity management planning. Even simple additions to existing inspection checklists at sites of excavation damage, such as documenting the type of coating present on affected steel service lines, could help a utility passively document key characteristics of its asset base in greater detail.

Although this case study focuses on natural gas service pipelines, the fundamental approach highlights opportunities for other large engineered systems, such as electric power and water distribution networks and municipal stormwater management systems. As the climate changes and the energy system transitions to new modes of operation, these systems may encounter stresses on system components that were not anticipated in their initial design. In these cases, various forms of analysis, including engineering simulation modeling, can provide useful insights to inform maintenance and operational procedures. In many instances, such an analysis may require additional data collection beyond what has already been gathered for administrative purposes.

This method has several important limitations. If locations of excavation damage are strongly correlated with asset characteristics of interest, for example, if excavation activity is higher in a region with a disproportionate share of plastics of a given type, this could introduce significant bias into estimated prevalence across the asset base. Changes over time in the rate of excavation damage would not necessarily bias the validity of the results but only if the change is not correlated with asset characteristics of interest. Smaller utilities with fewer instances of excavation damage may require a longer time series to collect enough data to meaningfully apply this method to their asset base.

As in this study, many important forms of analysis, for example, assessing the rough cost of retrofits for various engineered systems, will often require only data from a representative sample of assets. For aboveground assets, it is easier to gain direct access. If the cost of measurement is low, it may be possible to simply collect measurements at a random sample of the assets in question. For underground or underwater assets or assets embedded within the walls or foundations of buildings, the cost and disruption of collecting a truly random sample of in situ measurements is likely prohibitive. In these cases, it may be possible to identify a type of exogenous incident that is uncorrelated with the measured quantities and provides direct access to the asset to enable measurement.

The approaches outlined in this paper will hopefully inform data collection and analysis procedures to help ensure a safe, well-understood transition for long-lived infrastructures undergoing rapid operational change in an uncertain energy future.

## Supporting Information Available

Further data description; leak incidence rates by region; leaks in other components by cause; all leak causes; additional asset composition summary statistics; and evaluation of statistically significant differences in median asset age (PDF)
