# Supplementary material for: Low-Cost Representative Sampling for a Natural Gas Distribution System in Transition
Source: ACS Omega. 2022 Nov 23;7(48):43973–80. doi: 10.1021/acsomega.2c05314 (PMC9730304; doi:10.1021/acsomega.2c05314)
Supplement: Supplementary file 1 — ao2c05314_si_001.pdf [file ao2c05314_si_001.pdf]

Supporting information for: Low-cost representative sampling for a natural gas distribution system in transition

Evan D. Sherwin[1,*], Ernest Lever[2], Adam R. Brandt[1]

[1] Department of Energy Science & Engineering, Stanford University, 367 Panama St., Stanford, CA 94305.

[2] Gas Technology Institute. 1700 S. Mount Prospect Rd. Des Plaines, IL 60018.

[*] Correspondence: evands@stanford.edu

S1 Further data description

These datasets were collected by our partner utility. The asset database is a digital record of all assets in the system, maintained by the utility to reflect the status of the system. It includes information about the location, design specifications, and installation of the asset.

The leak database is generated through routine leak surveys, primarily conducted with handheld methane sensors, as well as leaks reported by companies interfacing with utility assets or called in by the public, together with a small number of leaks identified through vehicle surveys. Excavation damage leaks are generally reported by the damaging party or are identified by utility personnel.

The supplementary spreadsheet "Field descriptions Sherwin et al.xlsx" lists all fields in the leak and service line databases, as well as matched fields used in this analysis for comparing installation year, pipeline diameter, pressure rating, and division. Due to the data use agreement with our partner utility, we cannot share the underlying raw data.

The databases do not reliably allow one-to-one matching between individual leaks and the corresponding asset in the database. As a result, this study focuses on statistically comparing characteristics observed for both service lines affected by excavation damage leaks and service lines it the full population.

S1.1 Components considered in this analysis

This work primarily focuses on leaks in service lines. However, the leaks database also includes leaks from other common natural gas distribution system components. These include tee caps, service valves, service pipelines, above-ground and below-ground risers, regulators, main pipelines, and main valves. In some instances, leaks from transmission assets are included in the service leak database. Figure S1 provides the breakdown of leaks by source component both for the full database and for leaks recorded from 2009 onward.

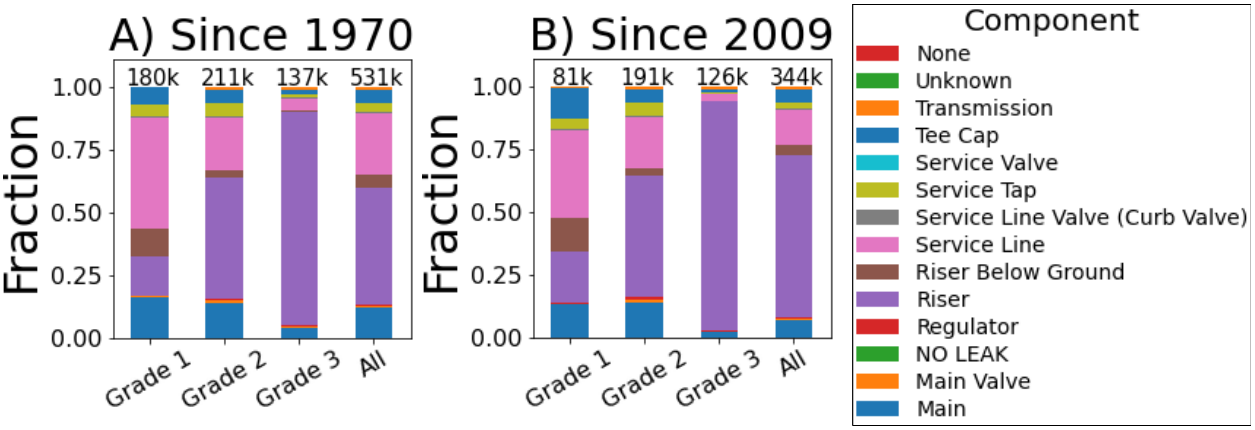

Figure S1. Leak breakdown by component and grade. A) includes all leaks in the full database since 1970. B) includes only leaks from 2009 onward. Total leak counts are listed above each bar.

S2 Leak incidence rates by region

Table S1 through Table S4 examine variation in leak rates for plastic and steel service lines across divisions within the utility's service territory. Columns include total service line counts, average service line length, total service line length, total excavation-related leaks from 2009-2019 (the period in which excavation incidence has been roughly stationary), and leaks per kilometer of service line. Table S1 and

Table S2 represent plastic service lines, while Table S3 and Table S4 represent steel service lines. Division names are anonymized.

Table S1 and Table S3 show only divisions with at least 1,000 km of plastic service line or 500 km of steel service line, respectively. These represent 97% and 99.9% of all plastic or steel service lines, respectively, in the utility's full service territory. These two sets of divisions are not identical between plastic and steel materials.

Table S2 and Table S4 show divisions with fewer than 1,000 km of plastic service line or 500 km of steel service line, respectively. In some cases, these divisions have much higher excavation leak incidence rates per kilometer of service line. This is likely due, at least in part, to accounting differences across the service line and leak databases. The precise names and boundaries of divisions may change over time and may not be consistent across databases, potentially introducing artifacts into analyses of leak incidence per length of service line installed. We do not have access to the precise division boundaries or their evolution over time.

Table S1. Leak incidence by division for plastic service lines across all divisions with at least 1,000 km of plastic service line (97.09% of total plastic service lines in the utility's territory) from 2009 to early 2019. Division names are anonymized. To compute approximate leaks per km per year, divide leaks/km by 10 years.

Division ID	Asset count	Average length [m]	Length [km]	Excav. leaks	Excav. leaks/km
1	39,008	100	1,191	301	0.253
4	109,094	98	3,247	603	0.186
5	122,759	101	3,771	73	0.019
7	73,158	100	2,224	424	0.191
8	108,640	100	3,306	858	0.260
10	55,951	104	1,781	209	0.117
12	271,169	98	8,105	739	0.091
13	147,455	67	3,023	1426	0.472
14	132,094	100	4,008	518	0.129
15	81,216	94	2,324	465	0.200
16	68,529	99	2,063	440	0.213
17	108,242	100	3,294	306	0.093
18	161,024	101	4,954	493	0.100

Table S2. Leak incidence by division for plastic service lines across all divisions with at least 1,000 km of plastic service line (2.81% of total plastic service lines) from 2009 to early 2019. The small length of plastic service line in these areas may be due to differences in accounting practices across assets and leaks. Division names are anonymized. To compute approximate leaks per km per year, divide leaks/km by 10 years.

Division ID	Asset count	Average length [m]	Length [km]	Excav. leaks	Excav. leaks/km
2	22,766	99	686	779	1.13
3	410	68	9	1118	130.79
6	5,222	109	174	364	2.09
9	228	76	5	30	5.70
11	14,280	97	422	290	0.69

Table S3. Leak incidence by division for steel service lines across all divisions with at least 500 km of steel service line (99.9987% of total steel service lines) from 2009 to early 2019. Division names are anonymized. To compute approximate leaks per km per year, divide leaks/km by 10 years.

Division ID	Asset count	Average length [m]	Length [km]	Excav. Leaks	Leaks/Km
1	27,928	97	825	48	0.058
2	36,947	101	1,140	58	0.051
4	67,677	95	1,969	62	0.032
5	57,220	92	1,596	3	0.002
7	27,588	93	782	36	0.046
8	90,973	101	2,806	78	0.028
10	24,110	103	753	11	0.015
12	145,762	97	4,308	109	0.025
13	24,914	88	670	207	0.309
14	119,948	93	3,382	41	0.012
16	24,526	98	734	42	0.057
17	31,004	96	908	32	0.035
18	58,892	97	1,742	48	0.028

Table S4. Leak incidence by division for steel service lines across all divisions with at least 500 km of steel service line (0.0013% of total steel service lines) from 2009 to early 2019. The small length of steel service line in these areas may be due to differences in accounting practices across assets and leaks. Division names are anonymized. To compute approximate leaks per km per year, divide leaks/km by 10 years.

Division ID	Asset count	Average length [m]	Length [km]	Excav. leaks	Excav. leaks/km
3	16	49	0.2	72	303.993
6	3,184	104	101.4	23	0.227
9	5	43	0.1	5	76.525
11	6,233	99	188.4	30	0.159
15	16,534	96	485.4	56	0.115

S3 Leaks in other components by cause

Figure S2 and Figure S3 present the composition and number of leaks by cause for plastic and steel risers and distribution mains. Note that the leak composition is quite different for these two sets of components than for distribution service lines. Risers tend to have more pipe dope-related leaks, particularly for steels. Excavation damage and corrosion are even more dominant for plastic and steel distribution mains, respectively, than for service lines.

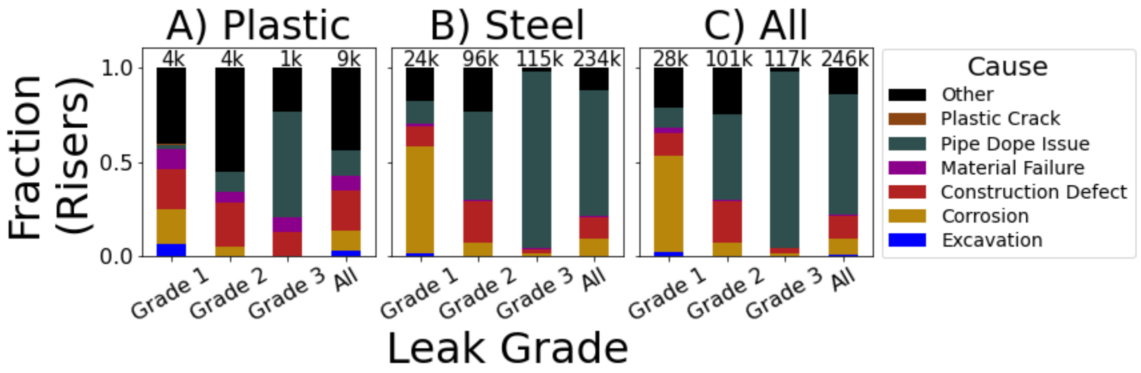

*Figure S2. Breakdown of selected leak types by grade for risers by material, including A) Plastic risers, B) Steel risers, and C) All risers, regardless of material. Total leak counts for each category are presented at the top of each bar. Grade 1 leaks are the most severe and require the most immediate attention. Grade 1 leaks pose the highest safety risk, while Grade 3 pose the lowest. Pipe dope issues are the most prevalent type of leak overall for these components and are typically Grade 2 or 3 leaks. *"Excavation" uses the "Digin/Excavation" cause code from the database. See the SI, Section S4 for all causes. Corrosion combines the "Atmospheric Corrosion" and "External Corrosion" cause codes. Grade 2 includes both grades 2 and 2+ (a sub-category of grade 2 leaks, now discontinued by this utility).*

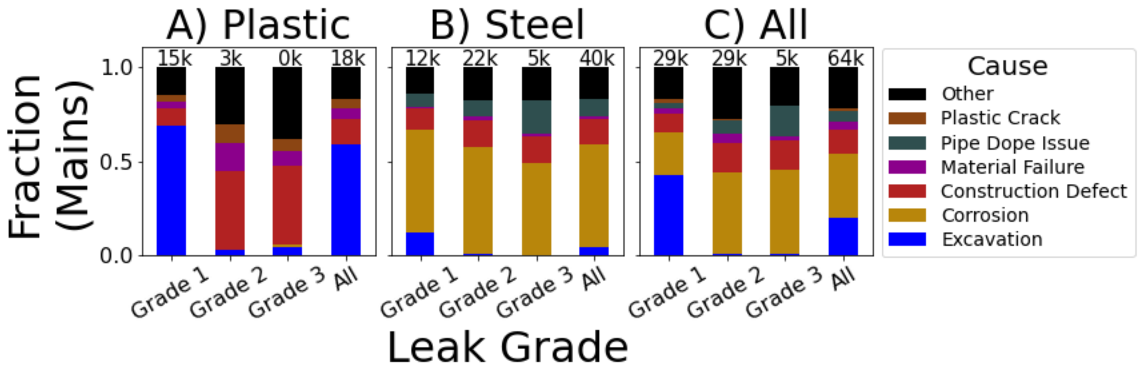

*Figure S3. Breakdown of selected leak types by grade for distribution main pipelines by material, including A) Plastic mains, B) Steel mains, and C) All mains, regardless of material. Excavation damage is the primary cause of leaks in plastic distribution mains, while corrosion is the dominant cause for steel mains. Total leak counts for each category are presented at the top of each bar. *"Excavation" uses the "Digin/Excavation" cause code from the database. See the SI, Section S4 for all causes. Corrosion combines the "Atmospheric Corrosion" and "External Corrosion" cause codes. Grade 2 includes both grades 2 and 2+ (a sub-category of grade 2 leaks, now discontinued by this utility).*

S4 All leak causes

Table S5. Full breakdown of leaks by cause (leakcausedesc). Includes leak counts for the full database from 1970 on for all components. The total number of leaks is 413 below the total count in the database (0.08%) due to null cause codes.

Leak cause	Count	Percentage
Atmospheric Corrosion	22,230	4.19%
Cast Iron Fracture	405	0.08%
Compression Coupling	848	0.16%
Construction Defect	81,954	15.45%
Damage by Earth Movement	7,156	1.35%
Damage by Electrical Facility	269	0.05%
Damage by Heavy Rain/Flood	167	0.03%
Damage by Third Party (non-digin)	3,163	0.60%
Deliberate Acts/Vandalism	89	0.02%
Digin/Excavation	63,566	11.98%
Earthquake	39	0.01%
Equipment Malfunction	1,649	0.31%
External Corrosion	61,967	11.68%
Fire or Explosion on Company Facility	67	0.01%
Fire or Explosion on Customer Facility	208	0.04%
Incorrect Operation	2,757	0.52%
Internal Corrosion	1,074	0.20%
Leak	4	0.00%
Leak - Unknown	48	0.01%
Lightning	6	0.00%
Material Failure	21,037	3.97%
No/Deteriorated Pipe Dope	170,198	32.09%
Other	75,035	14.15%
Other Natural Forces	921	0.17%
Plastic Crack Failure	5,942	1.12%
Plastic Embrittlement	1,653	0.31%
Previously Damaged	780	0.15%
Rodent	243	0.05%
Root Damage	815	0.15%
Stress Corrosion Cracking	421	0.08%
Unknown (Replaced Facility)	2,707	0.51%
Vehicle	663	0.13%
Weld Failure	2,318	0.44%
None	0	0.00%
Total	530,399	100.00%

S5 Additional asset composition summary statistics

S5.1 Service lines

Table S6. Service line count by diameter and material.

Diameter [in]	Plastic	Steel
0.25	4096	1
0.5	1294006	4577
0.75	404	682904
1	183444	5352
1.25	23590	42656
1.5	16	620
10	8	22
2	8799	4968
3	760	794
4	416	347
6	32	71
8	4	9
Unknown	5670	21119

Table S7. Service line length [meters], average and standard deviation.

Diameter [in]	Steel avg	Steel stddev	Plastic avg	Plastic stddev
0.25	101	nan	nan	nan
0.5	83	37	100	26
0.75	98	31	96	29
1	95	43	94	57
1.25	97	61	97	67
1.5	88	74	113	121
10	92	28	95	30
12	97	18	80	25
2	103	137	147	214
2.5	110	211	100	26
3	106	171	156	313
4	138	280	275	540
6	218	326	383	477
8	260	387	174	152
Unknown	46	43	44	31

Table S8. Steel service line pressure classification

Diameter [in]	All	0.5	0.75	1	1.25	2
Low pressure	24735	15	317	297	21681	1459
Sub-high pressure	47340	107	41687	594	3726	461
High pressure	691384	4455	640899	4461	17249	3047
Sub-high pressure	2	nan	1	nan	nan	1

Table S9. Plastic service line pressure classification (plastic pipelines are generally not coated).

Diameter [in]	All	0.5	0.75	1	1.25	2
Low pressure	25582	408	2	19788	3975	1186
Sub-high pressure	55651	47900	144	6471	687	354
High pressure	1440008	1245697	258	157183	18928	7258
Sub-high pressure	4	1	nan	2	nan	1

Table S10. Steel coating type.

	All	0.5	0.75	1	1.25	2
Asphalt	2	nan	nan	nan	2	nan
Bare	36	nan	27	2	7	nan
Double Wrapped	10210	4	8690	3	159	22
Fusion Bond Epoxy	1	nan	nan	nan	1	nan
Hot Applied Tape	1	nan	1	nan	nan	nan
Not Applicable	472	9	315	13	88	26
Paint	1	nan	nan	1	nan	nan
Plastic Coated	5029	20	4800	3	151	33
Single Wrapped	396323	2515	352386	3284	31689	3804
Somastic	62	nan	61	nan	nan	nan
Unknown	351222	2029	316524	2046	10559	1081
X-Tru-Coat	102	nan	100	nan	nan	2

Table S11. Installation year [years].

Diameter [in]	Steel avg	Steel stddev	Plastic avg	Plastic stddev
0.25	1970	nan	nan	nan
0.5	1971	21	1996	6
0.75	1959	9	1992	11
1	1953	17	1975	14

1.25	1955	16	1998	13
1.5	1937	20	1993	11
2	1968	16	1995	11
2.5	1953	nan	1993	10
3	1974	17	1999	10
4	1974	15	2004	8
6	1985	14	2014	1
8	1975	22	1995	11
10	1959	10	1981	8
12	1962	22	nan	nan
Unknown	1968	17	1988	7

S5.2 Regulators

Table S12. Unit pressure rating.

Pressure rating	count
Low pressure	261
Sub-high pressure	102
High pressure	2413
Transmission	2895

Table S13. Inlet maximum operating pressure [PSI] by unit pressure rating.

Pressure rating	avg	stddev
Low pressure	50	40
Sub-high pressure	154	185
High pressure	395	220
Transmission	450	255

Table S14. Outlet maximum operating pressure [PSI] by unit pressure rating.

Diameter [in]	Avg	Stddev
Low pressure	12	3
Sub-high pressure	21	11
High pressure	54	10
Transmission	145	177

Table S15. Above/below ground by unit pressure rating.

Diameter [in]	count	Below ground
Low pressure	8	253
Sub-high pressure	15	87
High pressure	493	1799
Transmission	125	570

Table S16. Number of stepdowns.

Diameter [in]	count
1	5263
2	332
3	75
4	1

Table S17. Installation year.

Diameter [in]	avg	stddev
Low pressure	1987	20
Sub-high pressure	1988	24
High pressure	1994	19
Transmission	1981	20

Table S18. Number of regulators.

# regulators	Count
0	7
1	278
2	970
3	229
4	694
5	12
6	60
7	1
8	9
10	4
12	1
17	1
Nan	3405

S5.3 Non-controllable fittings

Table S19. Material.

	Count
Plastic	437656
Steel	204311
Unknown	45656

Table S20. Joint type.

Joint type	All	Plastic	Steel
Butt Fusion	6695	6551	57
Caulked	8	5	3
Compression	48	34	10
Electrofusion	12971	12653	34
Flanged	77	56	21
Fused	179	175	4
Mechanical	1524	1161	358
Screwed	91	18	71
Socket Fusion	9647	9251	50
Stab	25174	25118	37
UNK	626264	381646	199362
Welded	5380	988	4304

Table S21. Installation year.

Material	avg	stddev
Plastic	2000	15
Steel	1968	20
Unknown	1987	18

Table S22. First Diameter (out of up to 4 diameters per unit) by material.

Diameter [in]	count	Plastic	Steel
0.25	163	132	8
0.38	2	nan	2
0.5	39936	35276	476
0.75	25172	1903	4957
1	77128	71933	474
1.25	72512	56799	12107
1.5	99	18	50
10	881	23	833
12	1753	62	1614
14	3	nan	3
16	988	49	882
18	38	4	34
2	317723	200486	109796
2.5	69	1	65
20	299	12	260
22	43	1	41
24	595	21	570
26	69	1	66
3	36913	12199	23243
3.5	3	nan	3
30	171	13	158
32	17	1	16
34	96	4	92
36	107	4	101
4	77832	46411	27833
40	2	nan	2
42	10	1	9
5	23	nan	21
5.5	1	nan	1
6	22006	8921	11280
8	7377	2304	4615
UNK	5854	1017	4596
None	173	60	103

S5.4 Controllable fittings

Table S23. Material.

Material	Count
Plastic	31080
Steel	300420
Unknown	16683

Table S24. Joint type by material type.

Joint type	All	Plastic	Steel
Butt Fusion	34	30	4
Compression	1	1	nan
Electrofusion	227	209	15
Flanged	14	1	11
Fused	21	13	8
Mechanical	45	27	18
Screwed	56	1	54
Socket Fusion	35	26	9
Stab	16	10	5
UNK	339055	30742	294794
Welded	8735	20	5502

Table S25. Installation year.

Material	avg	stddev
Plastic	1993	12
Steel	1970	20
Unknown	1987	14

Table S26. First Diameter (out of up to 4 diameters per unit) by material.

Diameter [in]	All	Plastic	Steel
0.25	5	nan	5
0.5	1821	60	1707
0.75	30445	8	30353
1	554	155	287
1.25	9784	2271	5874
1.5	17	2	14
10	1068	3	1035
12	1811	8	1696
14	1	nan	1
16	763	9	709
18	37	nan	37
2	171936	17781	146986
2.5	69	1	65
20	233	nan	224
22	28	nan	24
24	422	3	398
26	60	nan	60
3	38012	2197	34043
3.5	5	nan	5
30	78	3	74
32	6	nan	6
34	226	1	225
36	96	nan	96
4	53131	7176	42538
42	10	nan	10
5	27	nan	25
5.5	1	nan	1
6	17610	1167	14895
8	6759	172	6066
Unknown	13063	48	12821
None	161	15	140

S5.5 Excess flow valves

Table S27. Material.

Material	count
Plastic	312347
Steel	104
Unknown	46

Table S28. Valve size by material.

Valve size [in]	count	Plastic	Steel
0.5	160637	160613	nan
0.75	122	59	63
1	140164	140149	3
1.25	1231	1229	2
2	196	196	nan
Unknown	950	946	2
None	9197	9155	34

Table S29. Installation year by material

Material	avg	stddev
Plastic	2011	8
Steel	2016	3
Unknown	2013	4

S6 Evaluation of statistically significant differences in median asset age

We apply a Monte Carlo simulation to assess the statistical significance of the difference between the median age of all plastic and steel service lines in the database and those assets affected by excavation damage since 2009. For all four datasets, we randomly draw a sample of service line assets (with replacement) equal in size to the number of excavation damage incidents for that material, plastic (12,468) or steel (1,345). We then repeat this process 1,000 times and compute the median service line installation year of each of these 1,000 iterations. The minimum and maximum of these simulated median installation years form the 99.9% confidence interval for the median installation years of the selected dataset for a sample of the selected size. We find that the 99.9% confidence interval for the median installation year of all plastic service lines is 1991-1995, while for plastic service lines affected by excavation damage, it is 1982-1986. These ranges do not overlap, confirming that the observed difference in median age is statistically significant across the two datasets. For steel service lines, these ranges are 1959-1960 and 1955-1958, respectively. Thus, the difference between median installation years for steel service lines are also statistically significant, although with a smaller gap of only 2-6 years.
